# Soliton dynamics and stability in resonant nonlinear Schrödinger systems with cubic quintic effects via enhanced modified extended tanh function method

**DOI:** 10.1038/s41598-025-27692-5

**Published:** 2025-12-01

**Authors:** Amany Tarek, Hamdy M. Ahmed, Niveen Badra, Islam Samir

**Affiliations:** 1 Department of Physics and Mathematics Engineering, Faculty of Engineering, Ain Shams University, Cairo, Egypt; 2 Department of Physics and Engineering Mathematics, Higher Institute of Engineering El Shorouk Academy, Cairo, Egypt

**Keywords:** Soliton behavior, Higher-order dispersive media, Analytical wave localization, Stability analysis, Mathematics and computing, Optics and photonics, Physics

## Abstract

This study investigates solitary wave solutions of the three-dimensional, time-dependent nonlinear Schrödinger equation with cubic–quintic effects and a generalized Kudryashov-type self-phase modulation term. By applying the improved modified extended tanh function method, it obtains a broad spectrum of analytic solutions. These include bright soliton solutions, dark soliton solutions, singular periodic solutions, singular solutions, Jacobi elliptic function solutions, and a Weierstrass elliptic doubly periodic function solution. A detailed analysis demonstrates how variations in system parameters control the amplitude, width, and qualitative dynamics of the solitary waves. A comprehensive linear stability examination of the equilibrium points further reveals that parameter changes determine the emergence and disappearance of equilibrium states, with phase portraits illustrating the associated dynamical scenarios. The objectives are to establish the effectiveness of improved modified extended tanh function method in handling higher-dimensional nonlinear models, to enrich the catalogue of available exact solutions. The findings confirm that the proposed method is highly effective in generating exact solutions and in capturing intricate nonlinear structures influenced by the generalized Kudryashov contribution. This work expands the range of available solitary wave solutions and highlights their structural diversity. The study provides new theoretical insights into the interplay between nonlinearity, stability, and wave evolution, thereby offering valuable contributions to nonlinear optics, plasma physics, and higher-dimensional wave propagation.

## Introduction

The remarkable advancements in optical communication technologies over the past few decades have continuously pushed the boundaries of information transmission capacity and speed^1–5^. At the heart of these innovations lies the fundamental challenge of managing signal degradation caused by linear effects, primarily chromatic dispersion, which leads to pulse broadening and limits achievable data rates over long distances. While conventional solutions involve periodic signal regeneration, this approach introduces complexity, cost, and noise accumulation. This inherent limitation has driven significant research into phenomena that can intrinsically overcome dispersion, leading to the profound concept of optical solitons^6,7^.

Optical solitons, with their remarkable ability to maintain their shape and integrity over long distances, have found important applications in high-speed optical communication, all-optical signal processing, ultrafast laser technology, pulse generation, sensing, quantum optics and information, as well as in photonic devices and optical computing. The nonlinear Schrödinger equation (NLSE) is crucial because it fundamentally describes how optical solitons form and propagate in nonlinear media such as optical fibers^8–11^. It provides exact analytical solutions that offer deep insights into soliton behavior and forms the foundation for developing advanced theoretical models and numerical simulations in nonlinear optics. Beyond optics, the NLSE is a universal equation that appears in diverse fields, including Bose–Einstein condensates and water waves, highlighting its broad significance in nonlinear wave physics.

Studying nonlinear partial differential equations (NPDEs) is essential because they accurately model complex phenomena across various scientific and engineering disciplines, where linear approximations are insufficient^12–16^. Obtaining their exact solutions is paramount, as these solutions provide fundamental insights into the physical processes, serve as benchmarks for validating numerical simulations, and reveal underlying dynamics such as the formation of solitary waves and other localized structures. Various methods are employed to find these exact solutions. These techniques include the unified method^17^, the modified Sardar sub equation method^18,19^, the new extended direct algebraic method^19^, the Hirota bilinear method^20^, the non-classical symmetry analysis^21^, the complete discriminant system method^22^, the generalized Arnous method^22^, the generalized exponential rational function method^23^, modified Jacobi elliptic function method^24^, the general projective Riccati method^25^, the solitary wave ansatz^26^, Runge–Kutta fourth-order technique^27^, dynamical system analysis^28^, and Lie symmetries^29^.

In this paper, we deal with the (3+1)-dimensional nonlinear Schrödinger equation (NLSE) incorporating a generalized Kudryashov self-phase modulation (SPM). This equation reads^30^:

1 $$\begin{aligned} & i~R_t-(m_1~R_{xx}+m_2~R_{yy}+m_3~R_{zz}+2~m_4~R_{xy}-2~m_5~R_{xz}-2~m_6~R_{yx})\nonumber \\ & \quad +(s_1~|R|^{-n}+s_2~|R|^{-2n}+s_3~|R|^{-3n}+s_4~|R|^{-4n}+w_1~|R|^{n}+w_2~|R|^{2n}+w_3~|R|^{3n}+w_4~|R|^{4n})~R=0,~n>1. \end{aligned}$$

The complex-valued wave profiles are represented by *R*(*x*, *y*, *z*, *t*), where *t* denotes the temporal variable and *x*, *y*, *z* are the spatial variables. The equation incorporates several constant coefficients that are crucial for describing the behavior of the optical system. Specifically, $m_b$ (for $b=1,2,\ldots ,6$) are the coefficients of chromatic dispersion (CD), while $s_c$ and $w_c$ (for $c=1,2,3,4$) are the coefficients of self-phase modulation (SPM). Furthermore, the nonlinearity index *n*, which is a positive integer, serves as a power-law parameter characterizing the medium’s nonlinearity. Consequently, in the extended NLSE featuring cross-spatio-dispersion and Kudryashov’s SPM, the coefficients $m_b$, $s_c$, and $w_c$ constitute the fundamental parameters that govern the propagation of optical waves in multidimensional space.

Understanding nonlinear wave dynamics in higher-dimensional systems is fundamental to advancing our comprehension of numerous physical phenomena. The cubic-quintic nonlinear Schrödinger (CQ-NLS) model is a critical extension of the standard NLS equation, vital for accurately describing the behavior of high-intensity light pulses in various media. While the cubic term accounts for self-focusing effects, the addition of the quintic term is crucial for providing a self-defocusing effect that prevents catastrophic collapse, thus allowing for the formation of stable, high-power solitons. This stability is the cornerstone of its importance and applications. For instance, in optical communications, these stable solitons are ideal for carrying data over vast distances without degradation. In laser physics, the model is fundamental to understanding and controlling the dynamics of ultra-short pulses. Furthermore, it finds applications in emerging fields like plasmonics and nanophotonics, where it helps describe solitary waves in nanoscale waveguides. By balancing competing nonlinearities, the CQ-NLS model provides a powerful theoretical framework for designing and optimizing next-generation optical and photonic devices.

   In recent years, several analytical approaches have been developed to construct soliton solutions of various forms of important equations in real life. To highlight the novelty of the present study, we provide a detailed comparison with recent related works in the literature. The generalized Davey-Stewartson equation was studied through the $exp(-\Phi (\xi ))$-expansion method, the first integral method, and the Sine-Gordon expansion^31^. The generalized second-order nonlinear Schrödinger equation with light-wave promulgation in an optical fiber was studied through the first integral method , the $exp(-\Phi (\xi ))$-expansion method, and the $\left( \frac{G'}{G^2}\right)$-expansion function method^32^, and (1 + 2)-dimensional chiral nonlinear Schrödinger’s equation was studied through the first integral method, and the Sine-Gordon expansion^33^

Table 1 summarizes the main differences between several well-known forms of the nonlinear Schrödinger equation (NLSE) and the present generalized model. This comparison emphasizes how the additional terms and parameters in our formulation extend the physical applicability and allow for a richer set of soliton solutions.

**Table 1:** Comparison between different forms of the nonlinear Schrödinger equation (NLSE) and the present model.

Model	Equation	Features / Remarks
Standard NLSE	$i R_{t} +\alpha ~ R_{xx} +\beta ~ |R|^{2} R = 0$	Describes fundamental soliton dynamics in 1D with cubic nonlinearity.
Cubic–Quintic NLSE	$i R_{t} +\alpha ~ R_{xx} + (a |R|^{2} + b |R|^{4}) R = 0$	Includes both focusing and defocusing effects; models more complex nonlinear media.
Higher-Dimensional NLSE	$i R_{t} + (R_{xx} + R_{yy} + R_{zz}) + |R|^{2} R = 0$	Extends NLSE to 2D/3D; captures spatial–temporal solitons (light bullets, plasma waves).
Present Model	$$\begin{aligned}&i R_{t} - (m_{1} R_{xx} + m_{2} R_{yy} + m_{3} R_{zz}\\&+ 2 m_{4} R_{xy} - 2 m_{5} R_{xz} - 2 m_{6} R_{yx}) \\&\quad + (s_{1} |R|^{-n} + s_{2} |R|^{-2n} + s_{3} |R|^{-3n}\\&+ s_{4} |R|^{-4n} + w_{1} |R|^{n} + w_{2} |R|^{2n}\\&+ w_{3} |R|^{3n} + w_{4} |R|^{4n}) R = 0,\quad n > 1 \end{aligned}$$	Generalized model with mixed higher-order nonlinearities and cross-derivative terms; suitable for anisotropic and strongly nonlinear systems.

This work employs the improved modified extended tanh function method (IMETFM) to derive novel solutions for the proposed model. The obtained solutions encompass a wide variety of wave profiles. Several other analytical approaches have also been applied to this model, such as the modified direct algebraic method (MDAM), and the generalized Kudryashov method^34^, the new extended direct algebraic method (NEDAM)^35^, Fan-extended sub equation method under different constraint conditions^36^, the generalized exponential rational function method (GERFM)^37^, the extended sinh-Gordon equation expansion^38,39^, the $\left( \frac{G'}{G^2}\right)$-expansion function method^34^, the new auxiliary equation method (NAEM)^39^. The methodology is presented in Section 2, while Section 3 describes the application of this method to investigate Eq. (1). Section 4 provides both 3D and 2D visual illustrations that demonstrate the characteristics of these solutions along with an interpretation of their physical significance. A detailed linear stability analysis is carried out in Section 5. Finally, the study concludes with a summary of the key findings.

## The proposed algorithm

In this section the improved modified extended tanh function (IMETF) approach is presented^9^.

Considering the following NLPDE:

2 $$\begin{aligned} Y(R,R_t,R_x,R_y,R_y,R_{xx},R_{txy},R_{zxy},....)=0, \end{aligned}$$

where the function *Y* is unknown function in *R*(*x*, *y*, *z*, *t*) and its partial derivatives.

To handle Eq. (2) using IMETF approach, a number of steps must be executed:

**Step(1)**: Using the ensuing transformation, Eq. (2) can be transformed into an ODE.

3 $$\begin{aligned} R(x,y,z,t)=R(\xi ), ~ \xi =\xi =\eta _1~x+\eta _2~y+\eta _3~z-\gamma ~t,~ \gamma \ne 0, \end{aligned}$$

$\gamma$ denotes the traveling wave’s velocity. Then, Eq. (2) becomes:

4 $$\begin{aligned} Y(R,R',R'',R''',....)=0. \end{aligned}$$

**Step(2)**: The solution of Eq. (4) based on this proposed technique is obtained as the following form:

5 $$\begin{aligned} R(\xi )=h_0+\sum _{i=1}^{k}{h_i \rho (\xi )^i+j_i \rho (\xi )^{-i}}, \end{aligned}$$

where $\rho (\xi )$ meets the extended Riccati equation:

6 $$\begin{aligned} \rho '(\xi )=\sqrt{q_0+q_1 ~ \rho (\xi )+q_2 ~ \rho ^2(\xi )+q_3~ \rho ^3(\xi )+q_4 ~ \rho ^4(\xi )}. \end{aligned}$$

**Step (3)**: Use the balancing principle to determine the positive integer value *k* in Eq. (5) from Eq. (4).

**Step(4)**: Applying the suggested solution in Eq. (5) with Eq. (6) into Eq. (4) and equating the coefficients of $\rho ^i(\xi )$ to zero for $i=0,1,2,..$, a system of non-linear equations is created.

**Step(5)**: Using Mathematica applications, the nonlinear system of equations generated in step (4) can be solved to determine the unidentified values $h_i,~j_i$ and $\gamma$.

**Step(6)**: When $q_0,q_1,q_2,q_3,q_4$ have different possible values, Eq. (6) different types of solutions.

**Step(7)**: In this final phase, we replace the resulted unknowns and the general solution of Eq. (6) into the proposed solution of Eq. (5) to obtain exact solutions for Eq. (2).

Over the years, numerous analytical techniques have been developed to derive soliton solutions of nonlinear partial differential equations. Classical methods such as the inverse scattering transform, Hirota’s bilinear method, and Darboux transformations^40^ provide elegant frameworks for constructing multi-soliton solutions, though they are mainly applicable to integrable systems. Direct algebraic schemes, including the tanh-function expansion, sine–cosine method, and Jacobi elliptic expansions, extend applicability to non-integrable systems, yet often yield only a limited subset of solutions. More recent approaches, such as the improved simple equation method and projective Riccati techniques, have further advanced the range of accessible solutions but still face restrictions in handling higher-dimensional equations with complex nonlinearities.

Several recent works have specifically addressed the role of higher-order nonlinearities and generalized refractive index laws. For instance, Genc et al.^41^ investigated cubic–quartic optical solitons using F-expansion schemes and highlighted their rich stability properties. Similarly, Parasuraman^42^ studied dark soliton evolution in birefringent fibers under the Kundu–Eckhaus framework, emphasizing the influence of inter-modal dispersion. More recently, Alngar et al.^43^ analyzed cubic–quartic solitons within Kudryashov’s framework and demonstrated how arbitrary nonlinear refractive index laws can significantly affect solitary wave dynamics. These contributions confirm that incorporating generalized self-phase modulation terms, such as Kudryashov-type forms, is a powerful tool for constructing broader families of exact solutions.

Despite these advances, there remain limitations in achieving systematic and computationally simple frameworks that yield a wide spectrum of solitary waves, particularly in higher-dimensional systems. The IMETFM, employed in this study, addresses these challenges by enabling the derivation of bright, dark, singular, and elliptic solutions under the generalized CQ-NLS framework with resonant effects. By systematically combining algebraic expansion with a generalized Riccati structure, the IMETFM provides both analytical flexibility and computational tractability. This positions the current work as a significant extension of the existing literature, offering both theoretical insights and practical relevance for nonlinear optics and wave propagation. In this work the IMETFM enabled the derivation of not only bright and dark soliton solutions, but also singular solitons, singular periodic solutions, Jacobi elliptic function solutions, Weierstrass elliptic doubly periodic solutions, rational forms, and exponential solutions. Many of these structures, such as the singular periodic and elliptic solutions, are difficult or unattainable through alternative methods.

Although the IMETFM has proven efficient in constructing diverse exact solutions, it still has some limitations. Being an ansatz-based scheme, it may overlook solution families that fall outside the assumed functional form. Moreover, most obtained solutions exist only under specific parameter constraints, which restricts their general applicability. Finally, while the method provides exact expressions, further numerical simulations are required to confirm the stability and physical relevance of these solutions in realistic scenarios.

## Results

Our goal in this part is to get the next form of solutions for Eq. (1):

7 $$\begin{aligned} R(x,y,z,t)=R(\xi ) e^{i(\omega ~t-\nu _1~ x-\nu _2~ y-\nu _3~ z+\theta )},~ \xi =\eta _1~x+\eta _2~y+\eta _3~z-\gamma ~t, \end{aligned}$$

in which $\gamma$ denotes the velocity of the solitonn along the *x*, *y*, and *z* axes, while the wave numbers are symbolized by $\nu _1$, $\nu _2$, and $\nu _3$. Additionally, $\theta$ stands for phase constant, and $\omega$ represents the soliton frequency. Substituting by Eq. (7) into Eq. (1) then separating into real and imaginary parts, we have:

The real component:

8 $$\begin{aligned} & s_1~ R(\xi )^{1-n}+s_2~ R(\xi )^{1-2n}+s_3~ R(\xi )^{1-3n} +s_4~ R(\xi )^{1-4n}+w_1~ R(\xi )^{1+n}+w_2~ R(\xi )^{1+2n}+w_3~ R(\xi )^{1+3n}\nonumber \\ & \quad +w_4~ R(\xi )^{1+4n}+(-\omega +m_1~ \nu _1^2+2m_4~ \nu _1 ~ \nu _2-2m_6~\nu _3~\nu _2+m_2~ \nu _2^2-2m_5~\nu _3~\nu _1+m_3~ \nu _3^2)~R(\xi )\nonumber \\ & \quad -R''(\xi )~(m_1~ \eta _1^2+2m_4~ \eta _1~\eta _2-2m_6~\eta _3~\eta _2+m_2~\eta _2^2-2m_5~\eta _1~\eta _3 +m_3~\eta _3^2)=0. \end{aligned}$$

The imaginary component:

9 $$\begin{aligned} & \gamma -2m_1~\nu _1~\eta _1+2m_6~\nu _2~\eta _3+2m_5~\nu _3~\eta _1+2m_6~\nu _1~\eta _2-2m_2~\nu _2~\eta _2\nonumber \\ & \quad -2m_4~(\nu _2~\eta _1+\nu _1~\eta _2)+2m_5~\nu _1~\eta _3-2m_3~\nu _3~\eta _3=0. \end{aligned}$$

Equalizing the coefficient of Eq. (9) to zero provides

10 $$\begin{aligned} \gamma =2\eta ~(m_1~\nu _1+m_4~\nu _2-m_5~\nu _3)+2\eta _2~(-m_6~\nu _1+m_2~\nu _2+m_4~\nu _2)+2\eta _3~(m_3~\nu _3-m_5~\nu _1-m_6~\nu _2). \end{aligned}$$

To implement the IMETF scheme on the real portion, the balance principle has to applied to get the integer *k*, by comparing the highest-order linear derivative term with the leading-order nonlinear term.

According to Eq. (9), the chain rule applied to the function $R(\xi ) = \sum _{i=-N}^{N} \alpha _{i} W(\xi )^{i}$ results in terms with dominant behavior of order $\mathbf {W^{N+2}}$ from the second derivative $R''$. Conversely, terms of leading order $\mathbf {W^{2N}}$ are produced by the nonlinear term $\mathbf {R^{1+4n}}$.

By equating the dominant powers of $W(\xi )$ in both terms, we get:

$$\begin{aligned} k+2=(1+4n)~k\quad \implies \quad k=\frac{1}{2n}. \end{aligned}$$,

so the following transformation can be applied on the real part to get an integer value for *k*:

11 $$\begin{aligned} R(\xi ) =U(\xi )^{\frac{1}{2n}}. \end{aligned}$$

Eq. (8) becomes:

12 $$\begin{aligned} & 4n^2~s_4+4n^2~s_2~U(\xi )+4n^2~(\Pi _2-\omega )~U(\xi )^2+4n^2~w_2~U(\xi )^3+4n^2~w_4~U(\xi )^4+(1-2n)~\Pi _1~U'(\xi )^2\nonumber \\ & \quad +2n~\Pi _1~U''(\xi )~U(\xi )=0, \end{aligned}$$

Where

13 $$\begin{aligned} & \Pi _1=-(m_1~\eta _1^2+2m_4~\eta _1~\eta _2+m_2~\eta _2^2-2m_5~\eta _1~\eta _3-2m_6~\eta _2~\eta _3+m_3~\eta _3^2), \end{aligned}$$

14 $$\begin{aligned} & \Pi _2= m_1~\nu _1^2+2m_4~\nu _1~\nu _2+m_2~\nu _2^2-2m_5~\nu _1~\nu _3-2m_6~\nu _2~\nu _3+m_3~\nu _3^2. \end{aligned}$$

Balancing $U(\xi )^4$ with $U''(\xi )~U(\xi )$, we get $k=1$ by the balancing procedure that was previously mentioned, $4k=k+2+k$. According to the proposed approach, the solution of Eq.(12) will be declared into the next form

15 $$\begin{aligned} U(\xi )=h_0+h_1 \rho (\xi )+j_1 \rho (\xi )^{-1}. \end{aligned}$$

Substituting by Eq. (15) and its Riccati equation Eq. (6) into Eq. (12) then equating all the coefficients of $\rho ^i$ to zero, a system of non-linear equations will be resulted that can be resolved using mathematica packages. The results will be as follow:

**Case 1**. $$q_0=q_1=q_3=0$$

**Result(1)**

$$q_2=\frac{q_4~(\omega +2n~\omega +6w_4~h_0^2-\Pi _2 -2n\Pi _2 )}{w_4~h_1^2},~ q_4=\frac{-4n^2~w_4~h_1^2}{(1+2n)\Pi _1},~ j_1=0, ~h_0 =\frac{-(1+2n)w_2}{4(1+n)w_4},$$

$${-22em} s_2=\frac{2(-1+n)~w_4~h_0~(2q_4~h_0^2+q_2~h_1^2)}{(1+2n)~q_4}.$$

The next bright soliton solution can then be elevated

16 $$\begin{aligned} R(x,y,z,t)= & \left[ -\frac{(1+2n)w_2}{4(1+n)w_4}+\sqrt{\frac{\frac{3(1+2n)^2~w_2^2}{8(1+n)^2~w_4}+(1+2n)(\omega -\Pi _2)}{w_4}}\right. \nonumber \\ & \left. \quad \operatorname {sech}\left( 2(\eta _1~x+\eta _2~y+\eta _3~z-\gamma ~t) \sqrt{\frac{n^2\left( \frac{3(1+2n)^2~w_2^2}{8(1+n)^2~w_4}+(1+2n)(\omega -\Pi _2)\right) }{(1+2n)~\Pi _1}}\right) \right] ^{\frac{1}{2n}}\nonumber \\ & \times e^{i(t \omega +\theta -\nu _1 x-\nu _2 y-\nu _3 z)} \nonumber \\ & ,{\frac{\frac{3(1+2n)^2~w_2^2}{8(1+n)^2~w_4}+(1+2n)(\omega -\Pi _2)}{w_4}>0,~ \frac{n^2\left( \frac{3(1+2n)^2~w_2^2}{8(1+n)^2~w_4}+(1+2n)(\omega -\Pi _2)\right) }{(1+2n)~\Pi _1}>0.} \end{aligned}$$

The next singular periodic solution can then be elevated

17 $$\begin{aligned} R(x,y,z,t)= & \left[ \frac{-(1+2n)~w_2}{4(1+n)~w_4}+\sqrt{\frac{\frac{3(1+2n)^2~w_2^2}{8(1+n)^2~w_4}+(1+2n)(\omega -\Pi _2)}{w_4}}\right. \nonumber \\ & \left. \times \sec \left( (\eta _1~x+\eta _2~y+\eta _3~z-\gamma ~t)~\sqrt{\frac{-n^2\left( \frac{3(1+2n)^2~w_2^2}{8(1+n)^2~w_4} +(1+2n)(\omega -\Pi _2)\right) }{(1+2n)~\Pi _1}}\right) \right] ^{\frac{1}{2n}}\nonumber \\ & \times e^{i(t \omega +\theta -\nu _1~ x-\nu _2~y-\nu _3~z)},\nonumber \\ & {\frac{\frac{3(1+2n)^2~w_2^2}{8(1+n)^2~w_4}+(1+2n)(\omega -\Pi _2)}{w_4}>0,~\frac{n^2 \left( \frac{3(1+2n)^2~w_2^2}{8(1+n)^2~w_4}+(1+2n)(\omega -\Pi _2)\right) }{(1+2n)~\Pi _1}<0.} \end{aligned}$$

**Case 2**. $$q_1=q_3=0$$

**Result(1)**

$$h_1=0,~h_0=\frac{-(1+2n)~w_2}{4~(1+n)~w_4},~q_2=\frac{n^2~((-1+n)~(1+2n)^2~w_2^3+16~(1+n)^3~s_2~w_4^2)}{2~(1+n)^2~(-1-n+2~n^2)~w_2~w_4~\Pi _1},~q_4=\frac{-(1+2n)~q_2^2~\Pi _1}{16~n^2~w_4~j_1^2}.$$

The next singular soliton is then elevated

18 $$\begin{aligned} R(x,y,z,t)= & \frac{1}{4}\left[ -\frac{(1+2n)w_2}{(1+n)w_4}\right. \nonumber \\ & \left. + \frac{\coth \left( \frac{1}{2}(\eta _1 x+\eta _2 y+\eta _3 z-\gamma t)\sqrt{-\frac{n^2((-1+n)(1+2n)^2w_2^3+16(1+n)^3s_2w_4^2)}{(1+n)^2(-1-n+2n^2)w_2w_4\Pi _1}}\right) }{\sqrt{\frac{(-1+n)(1+n)^2w_2w_4^2}{(-1+n)(1+2n)^2w_2^3+16(1+n)^3s_2w_4^2}}}\right] ^{\frac{1}{2n}}\nonumber \\ & \times e^{i(t \omega +\theta -\nu _1 x-\nu _2 y-\nu _3 z)}, \quad q_0=\frac{q_2^2}{4q_4} {,~\frac{n^2((-1+n)(1+2n)^2w_2^3+16(1+n)^3s_2w_4^2)}{(1+n)^2(-1-n+2n^2)w_2w_4\Pi _1}<0}\nonumber \\ & {,~\frac{(-1+n)(1+n)^2w_2w_4^2}{(-1+n)(1+2n)^2w_2^3+16(1+n)^3s_2w_4^2}>0.} \end{aligned}$$

The next singular periodic solution is then elevated

19 $$\begin{aligned} R(x,y,z,t)= & \frac{1}{4}~\left[ -\frac{(1+2n)~w_2}{(1+n)~w_4}+\frac{\cot \left[ \frac{1}{2}~(\eta _1~x+\eta _2~y+\eta _3~z-\gamma ~t)~\sqrt{\frac{n^2~((-1+n)~(1+2n)^2~w_2^3+16~(1+n)^3~s_2~w_4^2)}{(1+n)^2~(-1-n+2~n^2)~w_2~w_4~\Pi _1}}\right] }{\sqrt{-\frac{(-1+n)~(1+n)^2~w_2~w_4^2}{(-1+n)~(1+2n)^2~w_2^3+16~(1+n)^3~s_2~w_4^2}}}\right] ^{\frac{1}{2n}}\nonumber \\ & \times e^{i(t \omega +\theta -\nu _1 x-\nu _2 y-\nu _3 z)},~q_0=\frac{q_2^2}{4~q_4} {,~\frac{n^2~((-1+n)~(1+2n)^2~w_2^3+16~(1+n)^3~s_2~w_4^2)}{(1+n)^2~(-1-n+2~n^2)~w_2~w_4~\Pi _1}>0}\nonumber \\ & {,~\frac{(-1+n)~(1+n)^2~w_2~w_4^2}{(-1+n)~(1+2n)^2~w_2^3+16~(1+n)^3~s_2~w_4^2}<0.} \end{aligned}$$

The next Jacobi elliptic solutions can then be elevated

20 $$\begin{aligned} R(x,y,z,t)= & \frac{1}{8}~\left[ -~\frac{2~(1+2~n)~w_2}{(1+n)~w_4}+\right. \nonumber \\ & \left. \frac{\sqrt{2}~ j_1}{{{\,\textrm{cn}\,}}\left[ \frac{(\eta _1~x+\eta _2~y+\eta _3~z-\gamma ~t)~\sqrt{\frac{n^2~\left( (-1+n)~(1+2n)^2~w_2^3+16~(1+n)^3~s_2~w_4^2\right) }{(1+n)^2~(-1-n+2~n^2)~(-1+2p^2)~w_2~w_4~\Pi _1}}}{\sqrt{2}},p\right] ~\sqrt{\frac{(-1+n)~(1+n)^2~p^2~w_2~w_4^2~j_1}{(-1+2p^2)~\left( (-1+n)~(1+2n)^2~w_2^3+16~(1+n)^3~s_2~w_4^2\right) }}}\right] ^{\frac{1}{2n}}\nonumber \\ & \times e^{i(t \omega +\theta -\nu _1 x-\nu _2 y-\nu _3 z)},~q_0=\frac{q_2^2~p^2~(1-p^2)}{q_4~(2p^2-1)^2} {,~\frac{n^2~\left( (-1+n)~(1+2n)^2~w_2^3+16~(1+n)^3~s_2~w_4^2\right) }{(1+n)^2~(-1-n+2~n^2)~(-1+2p^2)~w_2~w_4~\Pi _1}>0}\nonumber \\ & {,~\frac{(-1+n)~(1+n)^2~p^2~w_2~w_4^2~j_1}{(-1+2p^2)~\left( (-1+n)~(1+2n)^2~w_2^3+16~(1+n)^3~s_2~w_4^2\right) }>0.} \end{aligned}$$

21 $$\begin{aligned} R(x,y,z,t)= & \frac{1}{8}~\left[ -\frac{2~(1+2~n)~w_2}{(1+n)~w_4}+\frac{j_1}{{{\,\textrm{dn}\,}}\left[ \frac{(\eta _1~x+\eta _2~y+\eta _3~z-\gamma ~t)~\sqrt{-\frac{n^2~((-1+n)~(1+2n)^2~w_2^3+16~(1+n)^3~s_2~w_4^2)}{(1+n)^2~(-1-n+2~n^2)~(-2+p^2)~w_2~w_4~\Pi _1}}}{\sqrt{2}},p\right] }\right. \nonumber \\ & \left. \sqrt{-\frac{(-1+n)^2~(1+n)^4~(1+2n)~p^2~w_2^2~w_4^3~j_1^2~\Pi _1}{n^2~(-2+p^2)((-1+n)~(1+2n)^2~w_2^3+16~(1+n)^3~s_2~w_4^2)^2}}\right] ^{\frac{1}{2n}}\nonumber \\ & \times e^{i(t \omega +\theta -\nu _1 x-\nu _2 y-\nu _3 z)},~q_0=\frac{q_2^2~(1-p^2)}{q_4~(2-p^2)^2}\nonumber \\ & {,~\frac{n^2~((-1+n)~(1+2n)^2~w_2^3+16~(1+n)^3~s_2~w_4^2)}{(1+n)^2~(-1-n+2~n^2)~(-2+p^2)~w_2~w_4~\Pi _1}<0}\nonumber \\ & {,~\frac{(-1+n)^2~(1+n)^4~(1+2n)~p^2~w_2^2~w_4^3~j_1^2~\Pi _1}{n^2~(-2+p^2)((-1+n)~(1+2n)^2~w_2^3+16~(1+n)^3~s_2~w_4^2)^2}<0.} \end{aligned}$$

22 $$\begin{aligned} R(x,y,z,t)= & \frac{1}{8}~\left[ -\frac{2~(1+2~n)~w_2}{(1+n)~w_4}+\frac{\sqrt{2}~j_1}{{{\,\textrm{sn}\,}}\left[ \frac{(\eta _1~x+\eta _2~y+\eta _3~z-\gamma ~t)~\sqrt{-\frac{n^2~((-1+n)~(1+2n)^2~w_2^3+16~(1+n)^3~s_2~w_4^2)}{(1+n)^2~(-1-n+2~n^2)~(1+p^2)~w_2~w_4~\Pi _1}}}{\sqrt{2}},p\right] }\right. \nonumber \\ & \left. \sqrt{\frac{(-1+n)~(1+n)^2~p^2~w_2~w_4^2~j_1^2}{(1+p^2)((-1+n)~(1+2n)^2~w_2^3+16~(1+n)^3~s_2~w_4^2)}}\right] ^{\frac{1}{2n}}\nonumber \\ & \times e^{i(t \omega +\theta -\nu _1 x-\nu _2 y-\nu _3 z)},~q_0=\frac{q_2^2~p^2}{q_4~(p^2+1)^2}\nonumber \\ & {,~\frac{n^2~((-1+n)~(1+2n)^2~w_2^3+16~(1+n)^3~s_2~w_4^2)}{(1+n)^2~(-1-n+2~n^2)~(1+p^2)~w_2~w_4~\Pi _1}<0}\nonumber \\ & {,~\frac{(-1+n)~(1+n)^2~p^2~w_2~w_4^2~j_1^2}{(1+p^2)((-1+n)~(1+2n)^2~w_2^3+16~(1+n)^3~s_2~w_4^2)}>0} \end{aligned}$$

**Result(2)**

$$j_1=0,~q_4=\frac{-4~n^2~w_4~h_1^2}{(1+2n)~\Pi _1},~h_0=\frac{-(1+2n)~w_2}{4~(1+n)~w_4},~q_2=\frac{q_4~(s_2+2~n~s_2+4~w_4~h_0^3-4~n~w_4~h_0^3}{2~(-1+n)~w_4~h_0~h_1^2}.$$

The next dark soliton solution is then elevated

23 $$\begin{aligned} R(x,y,z,t)= & \left[ \frac{-(1+2n)~w_2}{4~(1+n)~w_4}+\sqrt{\frac{(1+n)~\left( (s_2+2~n~s_2-\frac{(1+2n)^3~w_2^3}{16~(1+n)^3~w_4^2}+\frac{n~(1+2n)^3~w_2^3}{16~(1+n)^3~w_4^2}\right) }{(-1+n)~(1+2n)~w_2}} \right. \nonumber \\ & \left. \tanh \left( 2~(\eta _1~x+\eta _2~y+\eta _3~z-\gamma ~t)~\sqrt{-\frac{n^2~(1+n)~\left( s_2+2~n~s_2-\frac{(1+2n)^3~w_2~3}{16~(1+n)^3~w_4^2}+\frac{n~(1+2n)^3~w_2^3}{16~(1+n)^3~w_4^2}\right) ~w_4}{(-1+n)~(1+2n)~w_2~\Pi _1}}\right) \right] ^{\frac{1}{2n}} \nonumber \\ & \times e^{i(t \omega +\theta -\nu _1 x-\nu _2 y-\nu _3 z)},~q_0=\frac{q_2^2}{4~q_4} {,~\frac{(1+n)~\left( (s_2+2~n~s_2-\frac{(1+2n)^3~w_2^3}{16~(1+n)^3~w_4^2}+\frac{n~(1+2n)^3~w_2^3}{16~(1+n)^3~w_4^2}\right) }{(-1+n)~(1+2n)~w_2}>0}\nonumber \\ & \quad {,~\frac{n^2~(1+n)~\left( s_2+2~n~s_2-\frac{(1+2n)^3~w_2~3}{16~(1+n)^3~w_4^2}+\frac{n~(1+2n)^3~w_2^3}{16~(1+n)^3~w_4^2}\right) ~w_4}{(-1+n)~(1+2n)~w_2~\Pi _1}<0.} \end{aligned}$$

The next singular periodic solution is then elevated

24 $$\begin{aligned} R(x,y,z,t)= & \left[ \frac{-(1+2n)~w_2}{4~(1+n)~w_4}+\sqrt{-\frac{(1+n)~\left( (s_2+2~n~s_2-\frac{(1+2n)^3~w_2^3}{16~(1+n)^3~w_4^2}+\frac{n~(1+2n)^3~w_2^3}{16~(1+n)^3~w_4^2}\right) }{(-1+n)~(1+2n)~w_2}} \right. \nonumber \\ & \left. \tan \left( 2~(\eta _1~x+\eta _2~y+\eta _3~z-\gamma ~t)~\sqrt{\frac{n^2~(1+n)~\left( s_2+2~n~s_2-\frac{(1+2n)^3~w_2~3}{16~(1+n)^3~w_4^2}+\frac{n~(1+2n)^3~w_2^3}{16~(1+n)^3~w_4^2}\right) ~w_4}{(-1+n)~(1+2n)~w_2~\Pi _1}}\right) \right] ^{\frac{1}{2n}} \nonumber \\ & \times e^{i(t \omega +\theta -\nu _1 x-\nu _2 y-\nu _3 z)},~q_0=\frac{q_2^2}{4~q_4}{,~\frac{(1+n)~\left( (s_2+2~n~s_2-\frac{(1+2n)^3~w_2^3}{16~(1+n)^3~w_4^2}+\frac{n~(1+2n)^3~w_2^3}{16~(1+n)^3~w_4^2}\right) }{(-1+n)~(1+2n)~w_2}<0}\nonumber \\ & {,~\frac{n^2~(1+n)~\left( s_2+2~n~s_2-\frac{(1+2n)^3~w_2~3}{16~(1+n)^3~w_4^2}+\frac{n~(1+2n)^3~w_2^3}{16~(1+n)^3~w_4^2}\right) ~w_4}{(-1+n)~(1+2n)~w_2~\Pi _1}>0.} \end{aligned}$$

The next Jacobi elliptic solutions can then be elevated

25 $$\begin{aligned} R(x,y,z,t)= & \left[ \frac{-(1+2n)~w_2}{4~(1+n)~w_4}+\sqrt{2}~{{\,\textrm{cn}\,}}\left[ 2~\sqrt{2}~(\eta _1~x+\eta _2~y+\eta _3~z-\gamma ~t)\right. \right. \nonumber \\ & \left. \left. \sqrt{\frac{n^2~(1+n)~\left( s_2+2~n~s_2-\frac{(1+2n)^3~w_2^3}{16~(1+n)^3~w_4^2}+\frac{n~(1+2n)^3~w_2^3}{16~(1+n)^3~w_4^2}\right) ~w_4}{(-1+n)~(1+2~n)^2~(-1+2~p^2)~w_2~\Pi _1}},p\right] \right. \nonumber \\ & \left. \sqrt{\frac{(1+n)~p^2~\left( (s_2+2~n~s_2-\frac{(1+2n)^3~w_2^3}{16~(1+n)^3~w_4^2}+\frac{n~(1+2n)^3~w_2^3}{16~(1+n)^3~w_4^2}\right) }{(-1+n)~(1+2n)~(-1+2p^2)~w_2~h_1^2}}~h_1 \right] ^{\frac{1}{2n}} \nonumber \\ & \times e^{i(t \omega +\theta -\nu _1 x-\nu _2 y-\nu _3 z)}, ~q_0=\frac{q_2^2~p^2~(1-p^2)}{q_4~(2p^2-1)^2}{,~\frac{n^2~(1+n)~\left( s_2+2~n~s_2-\frac{(1+2n)^3~w_2^3}{16~(1+n)^3~w_4^2}+\frac{n~(1+2n)^3~w_2^3}{16~(1+n)^3~w_4^2}\right) ~w_4}{(-1+n)~(1+2~n)^2~(-1+2~p^2)~w_2~\Pi _1}>0}\nonumber \\ & {,~\frac{(1+n)~p^2~\left( (s_2+2~n~s_2-\frac{(1+2n)^3~w_2^3}{16~(1+n)^3~w_4^2}+\frac{n~(1+2n)^3~w_2^3}{16~(1+n)^3~w_4^2}\right) }{(-1+n)~(1+2n)~(-1+2p^2)~w_2~h_1^2}>0.} \end{aligned}$$

26 $$\begin{aligned} R(x,y,z,t)= & \left[ \frac{-(1+2n)~w_2}{4~(1+n)~w_4}+\frac{1}{2}~{{\,\textrm{dn}\,}}\left[ 2~\sqrt{2}~(\eta _1~x+\eta _2~y+\eta _3~z-\gamma ~t)\right. \right. \nonumber \\ & \left. \left. \sqrt{\frac{n^2~(1+n)~\left( s_2+2~n~s_2-\frac{(1+2n)^3~w_2^3}{16~(1+n)^3~w_4^2}+\frac{n~(1+2n)^3~w_2^3}{16~(1+n)^3~w_4^2}\right) ~w_4}{(-1+n)~(1+2~n)^2~(2-p^2)~w_2~\Pi _1}},p\right] \right. \nonumber \\ & \left. \sqrt{\frac{(1+2n)~p^2~\Pi _1}{n^2~(2-p^2)~w_4~h_1^2}}~h_1\right] ^{\frac{1}{2n}} \nonumber \\ & \times e^{i(t \omega +\theta -\nu _1 x-\nu _2 y-\nu _3 z)}, ~q_0=\frac{q_2^2~(1-p^2)}{q_4~(2-p^2)^2} {,~\frac{n^2~(1+n)~\left( s_2+2~n~s_2-\frac{(1+2n)^3~w_2^3}{16~(1+n)^3~w_4^2} +\frac{n~(1+2n)^3~w_2^3}{16~(1+n)^3~w_4^2}\right) ~w_4}{(-1+n)~(1+2~n)^2~(2-p^2)~w_2~\Pi _1}>0}\nonumber \\ & {\frac{(1+2n)~p^2~\Pi _1}{n^2~(2-p^2)~w_4~h_1^2}>0} \end{aligned}$$

27 $$\begin{aligned} R(x,y,z,t)= & \left[ \frac{-(1+2n)~w_2}{4~(1+n)~w_4}+\sqrt{2}~{{\,\textrm{sn}\,}}\left[ 2~\sqrt{2}~(\eta _1~x+\eta _2~y+\eta _3~z-\gamma ~t)\right. \right. \nonumber \\ & \left. \left. \sqrt{\frac{-n^2~(1+n)~\left( s_2+2~n~s_2-\frac{(1+2n)^3~w_2^3}{16~(1+n)^3~w_4^2}+\frac{n~(1+2n)^3~w_2^3}{16~(1+n)^3~w_4^2}\right) ~w_4}{(-1+n)~(1+2~n)^2~(1+p^2)~w_2~\Pi _1}},p\right] \right. \nonumber \\ & \left. \sqrt{\frac{(1+n)~p^2~\left( (s_2+2~n~s_2-\frac{(1+2n)^3~w_2^3}{16~(1+n)^3~w_4^2}+\frac{n~(1+2n)^3~w_2^3}{16~(1+n)^3~w_4^2} \right) }{(-1+n)~(1+2n)~(1+p^2)~w_2~h_1^2}}~h_1\right] ^{\frac{1}{2n}} \nonumber \\ & \times e^{i(t \omega +\theta -\nu _1 x-\nu _2 y-\nu _3 z)},~q_0=\frac{q_2^2~p^2}{q_4~(p^2+1)^2} {,~\frac{n^2~(1+n)~\left( s_2+2~n~s_2-\frac{(1+2n)^3~w_2^3}{16~(1+n)^3~w_4^2}+\frac{n~(1+2n)^3~w_2^3}{16~(1+n)^3~w_4^2}\right) ~w_4}{(-1+n)~(1+2~n)^2~(1+p^2)~w_2~\Pi _1}<0}\nonumber \\ & {,~\frac{(1+n)~p^2~\left( (s_2+2~n~s_2-\frac{(1+2n)^3~w_2^3}{16~(1+n)^3~w_4^2}+\frac{n~(1+2n)^3~w_2^3}{16~(1+n)^3~w_4^2}\right) }{(-1+n)~(1+2n)~(1+p^2)~w_2~h_1^2}>0.} \end{aligned}$$

**Case 3**. $$q_2=q_4=0$$, $$q_0\ne 0$$, $$q_1\ne 0$$, $$q_3>0.$$

**Result(1)**

$$q_0=\frac{-4n^2~w_4~j_1^2}{(1+2n)~\Pi _1},~ q_1=\frac{4~q_0~h_0}{j_1}-\frac{4n^2~w_2~j_1}{(1+n)~\Pi _1},~ q_3=\frac{h_0^2~(-3~q_1~j_1+4~q_0~h_0)}{j_1^3}+\frac{4n^2~s_2}{(-1+n)~j_1~\Pi _1},$$

$$h_0=\frac{1}{12}~\left( \frac{6-3(1+2n)~w_2}{(1+n)~c_4}+\sqrt{\frac{\left( -\frac{3}{1+n}\right) ^2~w_2^2+24(1+2n)~w_4~(\omega -\Pi _2 )}{w_4^2}}\right) .$$

The next Weierstrass elliptic doubly periodic function is elevated for Eq. (1)

28 $$\begin{aligned} R(x,y,z,t)= & \left[ \frac{1}{12}~\left( \frac{-3~(1+2n)~w_2}{(1+n)~w_4}+\tau \right) +j_1~/~\wp \left[ \frac{1}{12\sqrt{3}}~(\eta _1~x+\eta _2~y+\eta _3~z-\gamma ~t)~\sqrt{\frac{n^2}{j_1~\Pi _1}~\delta }\right. \right. \nonumber \\ & \left. \left. ,~\{\left( 576~w_4~j_1^2~\tau \right) ~/~((1+2n)~\delta ~)~,~(1728~w_4~j_1^2)~/~((1+2n)~\delta ~)\}~\right] +\right. \nonumber \\ & \left. h_1~ \wp \left[ \frac{1}{12\sqrt{3}}~(\eta _1~x+\eta _2~y+\eta _3~z-\gamma ~t)~\sqrt{\frac{n^2}{j_1~\Pi _1}~\delta }~,\right. \right. \nonumber \\ & \left. \left. \{\left( 576~w_4~j_1^2~\tau \right) ~/~((1+2n)~\delta ~)~,~(1728~w_4~j_1^2)~/~((1+2n)~\delta ~)\}~\right] \right] ^{\frac{1}{2n}}\nonumber \\ & \times e^{i(t \omega +\theta -\nu _1 x-\nu _2 y-\nu _3 z)}, q_0\ne 0, ~q_1\ne 0{,~\Pi _1>0.}, \end{aligned}$$

where

$$\tau = \sqrt{\frac{\left( 6-\frac{3}{1+n}\right) ^2~w_2^2+24(1+2n)~w_4~(\omega -\Pi _2 )}{w_4^2}}$$

$$\delta = \left( \frac{432~s_2}{-1+n}+\frac{1}{(1+n)^3~(1+2n)~w_4^2}~\left( -3~(1+2n)~w_2+(1+n)~w_4~\tau \right) ^2~(3~(1+2n)~w_2+2~(1+n)~w_4~\tau )~\right) .$$

**Case 4**. $$q_0=q_1=q_2=0$$

**Result(1)**

$$\begin{aligned} Q&= (1+n-2n^2)^3~w_2^3 \\&\quad +8\left( -4~(-1+n)^2~(1+n)^3~(1+2n)~s_2~w_4^2\right. \\&\quad \left. +\sqrt{(-1+n)^4~(1+n)^3~(1+2n)^2~s_2~w_4^2((-1+n)~(1+2n)^2~w_2^3+16~(1+n)^3~s_2~w_4^2)} \right) . \end{aligned}$$

$$\begin{aligned} h_0= & (w_2^2+2~n~w_2^2-3~n^2~w_2^2-4~n^3~w_2^2+4~n^4~w_2^2\\ & +w_2~Q^{1/3}+n~w_2~Q^{1/3}-2~n^2~w_2~Q^{1/3}+\frac{Q^{2/3}}{8~(-1+n)~(1+n)~w_4~Q^{1/3}}, \end{aligned}$$

$$j_1=0,~q_4=-\frac{4~n^2~w_4~h_1^2}{(1+2n)~\Pi _1}, q_3=-\frac{q_4~(s_2+2~n~s_2+4~w_4~h_0^3-4~n~w_4~h_0^3)}{3~(-1+n)~w_4~h_0^2~h_1}.$$

The next rational solution is elevated

29 $$\begin{aligned} R(x,y,z,t)= & \left[ \frac{A}{-8(1-n^2)w_4 Q^{1/3}} \right. \nonumber \\ & \left. + \frac{1}{12288 (1+2n) A^2 \Pi _1 \left( \frac{-8(1+n)n^2 Q^{1/3} (s_2(1+2n)-A)^3}{(1-n^2)^2} + \frac{n A^3}{128(1-n^2)^3 w_4^2 Q} \right) (\eta _1 x+\eta _2 y+\eta _3 z-\gamma t)^2} + \frac{16 n^2 w_4 h_1^2}{\Pi _1(1+2n)} \right] ^{\frac{1}{2n}}\nonumber \\ & \times e^{i(t \omega +\theta -\nu _1 x-\nu _2 y-\nu _3 z)},~q_4 \ne 0 {,(-1+n)^4~(1+n)^3~(1+2n)^2~ s_2~w_4^2((-1+n)~(1+2n)^2~w_2^3+16~(1+n)^3~s_2~w_4^2)>0}. \end{aligned}$$

The next exponential functions can be elevated

30 $$\begin{aligned} R(x,y,z,t)= & \left[ 3 (-1+n) \left( A\right) ^3+2 e^{\frac{64 (-1+n) n^2 (1+n)^2 w_4^2 Q^{2/3} \left( s_2+2 n~ s_2-\frac{\left( A\right) ^3}{B}\right) ~ s_1~ (\eta _1 x+\eta _2 y+\eta _3 z-\gamma t)}{3 (1+2 n)~ \left( A\right) ^2 ~\Pi _1 \sqrt{\frac{n^2~ w_4 ~s_1^2}{\Pi _1+2 n ~\Pi _1}}}} \left( 128 \left( 1-n^2\right) ^3~ s_2 ~w_4^2~Q+\right. \right. \nonumber \\ & \left. \left. n ~\left( 1-n^2\right) ^3~ s_2~ w_4^2 ~Q-\left( A\right) ^3+n~ \left( A\right) ^3\right) /\left( 24 (-1+n)^2~ (1+n) ~w_4~ Q^{1/3} ~\left( A\right) ^2 \right) \right] ^{\frac{1}{2n}} \nonumber \\ & \times e^{i(t \omega +\theta -\nu _1 x-\nu _2 y-\nu _3 z)},~q_4< 0,~w_2<0,~w_4<0{,~n<0,~s_2<0}, \end{aligned}$$

where:

$$A = w_2^2~(1 + 2n - 3n^2 - 4n^3 + 4n^4) + w_2 ~Q^{1/3}~(1 + n - 2n^2) + Q^{2/3},$$

$$\begin{aligned} B&= 128(1 - n^2)^3~ w_4^2 \left( -(1 + n - 2n^2)^3 ~w_2^3 + 32(-1 + n)^2~ (1 + n)^3 ~(1 + 2n)~ s_2~ w_4^2 \right. \\&\quad \left. - 8 \sqrt{(-1 + n)^4~ (1 + n)^3 ~(1 + 2n)^2~ s_2~ w_4^2~ \left( (-1 + n)~(1 + 2n)^2~ w_2^3 + 16(1 + n)^3 ~s_2~ w_4^2 \right) } \right) . \end{aligned}$$

**Case 5**. $$q_3=q_4=0$$

**Result(1)**

$$h_1=0,~q_0=-\frac{4 n^2~ w_4~ j_1^2}{(1+2 n) ~\Pi _1},~q_2=\frac{n^2~ \left( (1+2 n)^2~ w_2^3+\frac{16 (1+n)^3~ s_2~ w_4^2}{-1+n}\right) }{2 (1+n)^2 ~(1+2 n)~ w_2~ w_4~\Pi _1},~h_0=-\frac{(1+2 n)~ w_2}{4 (1+n)~ w_4}.$$

The next singular periodic solution is elevated

31 $$\begin{aligned} R(x,y,z,t)= & \left[ \frac{1}{4}~ \left( -\frac{(1+2 n)~ w_2}{(1+n)~ w_4}+\frac{\csc \left( \frac{(\eta _1 x+\eta _2 y+\eta _3 z-\gamma t) ~\sqrt{-\frac{n^2 \left( (1+2 n)^2~ w_2^3+\frac{16 (1+n)^3~s_2~ w_4^2}{-1+n}\right) }{(1+n)^2 ~(1+2 n)~ w_2~ w_4 ~\Pi _1}}}{\sqrt{2}}\right) }{\sqrt{\frac{(-1+n)~ (1+n)^2 ~w_2 ~w_4^2}{\left( -2-6 n+8 n^3\right) ~ w_2^3+32 (1+n)^3 ~s_2 ~w_4^2}}}\right) \right] ^{\frac{1}{2n}} \nonumber \\ & \times e^{i(t \omega +\theta -\nu _1 x-\nu _2 y-\nu _3 z)},~q_1=0 {,~\frac{n^2 \left( (1+2 n)^2~ w_2^3+\frac{16 (1+n)^3~s_2~ w_4^2}{-1+n}\right) }{(1+n)^2 ~(1+2 n)~ w_2~ w_4 ~\Pi _1}<0,~\frac{(-1+n)~ (1+n)^2 ~w_2 ~w_4^2}{\left( -2-6 n+8 n^3\right) ~ w_2^3+32 (1+n)^3 ~s_2 ~w_4^2}>0} \end{aligned}$$

The next singular soliton is elevated

32 $$\begin{aligned} R(x,y,z,t)= & \left[ \frac{1}{4} ~\left( -\frac{(1+2 n)~ w_2}{(1+n)~ w_4}+\frac{\sqrt{2} {{\,\textrm{csch}\,}}\left( \frac{(\eta _1 x+\eta _2 y+\eta _3 z-\gamma t)~ \sqrt{\frac{n^2 ~\left( (1+2 n)^2~ w_2^3+\frac{16 (1+n)^3~ s_2~ w_4^2}{-1+n}\right) }{(1+n)^2~ (1+2 n)~ w_2~ w_4 ~\Pi _1}}}{\sqrt{2}}\right] }{\sqrt{-\frac{(1+n)^2~w_2~ w_4^2 }{(1+2 n)^2 ~w_2^3+\frac{16 (1+n)^3 ~s_2 w_4^2}{-1+n}}}}\right) \right] ^{\frac{1}{2n}} \nonumber \\ & \times e^{i(t \omega +\theta -\nu _1 x-\nu _2 y-\nu _3 z)},~q_1=0{,~\frac{n^2 ~\left( (1+2 n)^2~ w_2^3+\frac{16 (1+n)^3~ s_2~ w_4^2}{-1+n}\right) }{(1+n)^2~ (1+2 n)~ w_2~ w_4 ~\Pi _1}>0} {,~\frac{(1+n)^2~w_2~ w_4^2 }{(1+2 n)^2 ~w_2^3+\frac{16 (1+n)^3 ~s_2 w_4^2}{-1+n}}<0} \end{aligned}$$

**Case 6**. $$q_0=q_1=0, ~q_4>0$$

**Result(1)**

$$j_1=0, h_0=\frac{h_1 ~\left( 4 n^2~ w_2~ h_1+q_3 ~\Pi _1+n~ q_3 ~\Pi _1\right) }{4 (1+n) ~q_4~ \Pi _1}, q_2=\frac{4 n^2~ \omega ~ h_1^2-6 q_4 ~h_0^2~ \Pi _1+3 q_3 ~h_0~ h_1~ \Pi _1-4 n^2~ h_1^2 ~\Pi _2}{h_1^2~ \Pi _1},$$

$$q_4=-\frac{4 n^2 ~w_4~ h_1^2}{(1+2 n)~ \Pi _1},~q_3=2\sqrt{q_2~ q_4}.$$

The next singular periodic solution is elevated The next dark soliton solution is elevated

33 $$\begin{aligned} R(x,y,z,t)= & \left[ \frac{1}{16 n ~\sqrt{w_4} ~\sqrt{q_4}~ \sqrt{\Pi _1}} ~ \sqrt{-1-2 n}~ \left( \frac{4 ~ n ~\sqrt{-1-2 n}~ w_2 ~\sqrt{q_4} \sqrt{\Pi _1}}{(1+n) ~\sqrt{w_4}}+2 q_3 ~\Pi _1+q_4 ~\Pi _1 \right. \right. \nonumber \\ & \left. \left. \sqrt{\frac{24 n^2 ~(1+2 n)~ w_2^2~ q_4+2 (1+n)^2~ w_4 ~(3 q_3^2 ~\Pi _1+32 n^2 ~q_4 (\omega -\Pi _2))}{(1+n)^2~ w_4~ q_4^2~ \Pi _1}} \left( 1 \right. \right. \right. \nonumber \\ & \left. \left. \left. +\tanh \left( \frac{(\eta _1 x+\eta _2 y+\eta _3 z-\gamma t)~ \sqrt{\frac{\frac{12 n^2 ~(1+2 n)~ w_2^2}{(1+n)^2~ w_4}+\frac{3 q_3^2~ \Pi _1}{q_4}+32 n^2~ (\omega -\Pi _2)}{\Pi _1}}}{4 \sqrt{2}}\right) \right) \right) \right] ^{\frac{1}{2n}} \nonumber \\ & \times e^{i(t \omega +\theta -\nu _1 x-\nu _2 y-\nu _3 z)}, ~w_4<0,\Pi _1>0 {,~n<0}\nonumber \\ & {,~\frac{24 n^2 ~(1+2 n)~ w_2^2~ q_4+2 (1+n)^2~ w_4 ~(3 q_3^2 ~\Pi _1+32 n^2 ~q_4 (\omega -\Pi _2))}{(1+n)^2~ w_4~ q_4^2~ \Pi _1}>0}\nonumber \\ & {\frac{\frac{12 n^2 ~(1+2 n)~ w_2^2}{(1+n)^2~ w_4}+\frac{3 q_3^2~ \Pi _1}{q_4}+32 n^2~ (\omega -\Pi _2)}{\Pi _1}>0.} \end{aligned}$$

## Illustrations of the soliton solutions

Here, the nature of some results, many extracted soliton results are highlighted in 2D and 3D graphs. Illustration (1) shows a visual modeling of a bright soliton from Eq. (16) by $$h_1=2, \omega =2, w_2=1.08, w_4=2,$$$$\eta _1=0.87, \eta _2=0, \eta _3=0,$$$$\gamma =-0, y=0, z=0, n=3, s_2=-1.315,$$$$s_4=-1.19, \Pi _1=2, \Pi _2=1.605$$. Illustration (2) shows a visual modeling of a dark soliton from Eq. (23) by $$h_1=-2, \omega =0.66, w_2=0.435, w_4=-0.775,$$$$\eta _1=0.925, \eta _2=-1.11, \eta _3=-0.86,$$$$\gamma =0.11, y=2.24, z=-4, n=2, s_2=1.71,$$$$s_4=-1.73, \Pi _1=1.135, \Pi _2=0.605$$. Illustration(3) shows a visual modeling of a singular periodic solution from Eq. (17) by $$h_1=2, \omega =2, w_2=1.08, w_4=2, \eta _1=-2,$$$$\eta _1=-2, \eta _2=-2, \eta _3=-2,$$$$\gamma =-0.625, y=0, z=0, n=2, s_2=1.315,$$$$s_4=1.495, \Pi _1=-1, \Pi _2=2$$. Illustration(4) shows a visual modeling of a singular soliton from Eq. (18) by $$j_1=0.785, \omega =-2, w_2=-2, w_4=-2,$$$$\eta _1=-2, \eta _2=-2, \eta _3=-2,$$$$\gamma =-0.225, y=2.18, z=-2.33, n=2, s_2=-2,$$$$s_4=-2, \Pi _1=0.8, \Pi _2=-2$$.

**Fig. 1:**
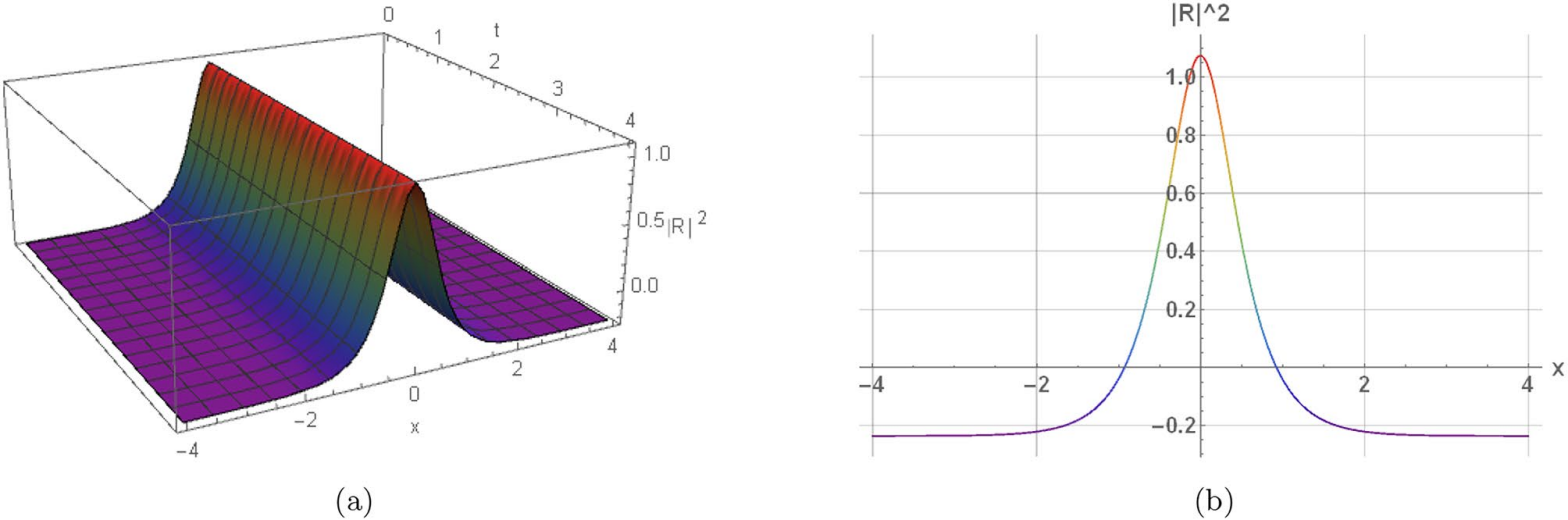
Graphical representation of a bright soliton of Eq. (16) by $$h_1=2, \omega =2, w_2=1.08, w_4=2, \eta _1=0.87, \eta _2=0, \eta _3=0,$$$$\gamma =-0, y=0, z=0, n=3, s_2=-1.315, s_4=-1.19, \Pi _1=2, \Pi _2=1.605$$.

**Fig. 2:**
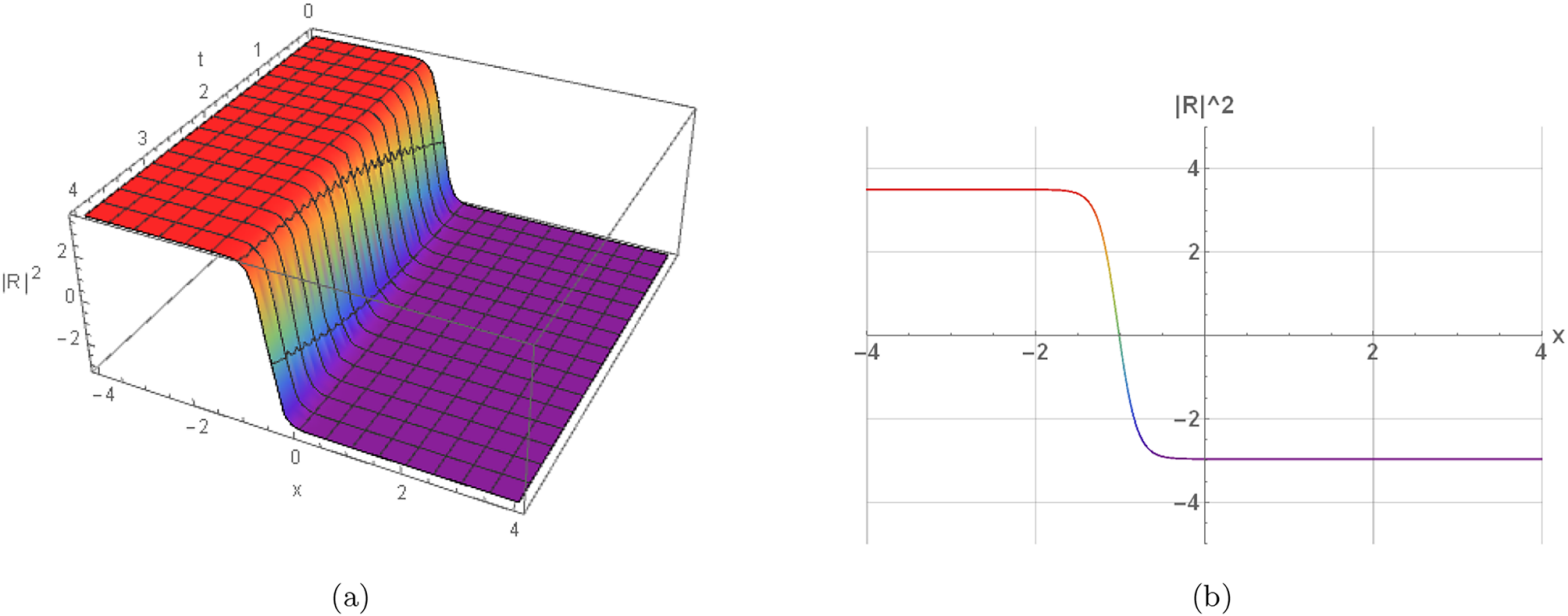
Graphical representation of a dark soliton of Eq. (23) by $$h_1=-2, \omega =0.66, w_2=0.435, w_4=-0.775, \eta _1=0.925, \eta _2=-1.11, \eta _3=-0.86,$$$$\gamma =0.11, y=2.24, z=-4, n=2, s_2=1.71, s_4=-1.73, \Pi _1=1.135, \Pi _2=0.605$$.

**Fig. 3:**
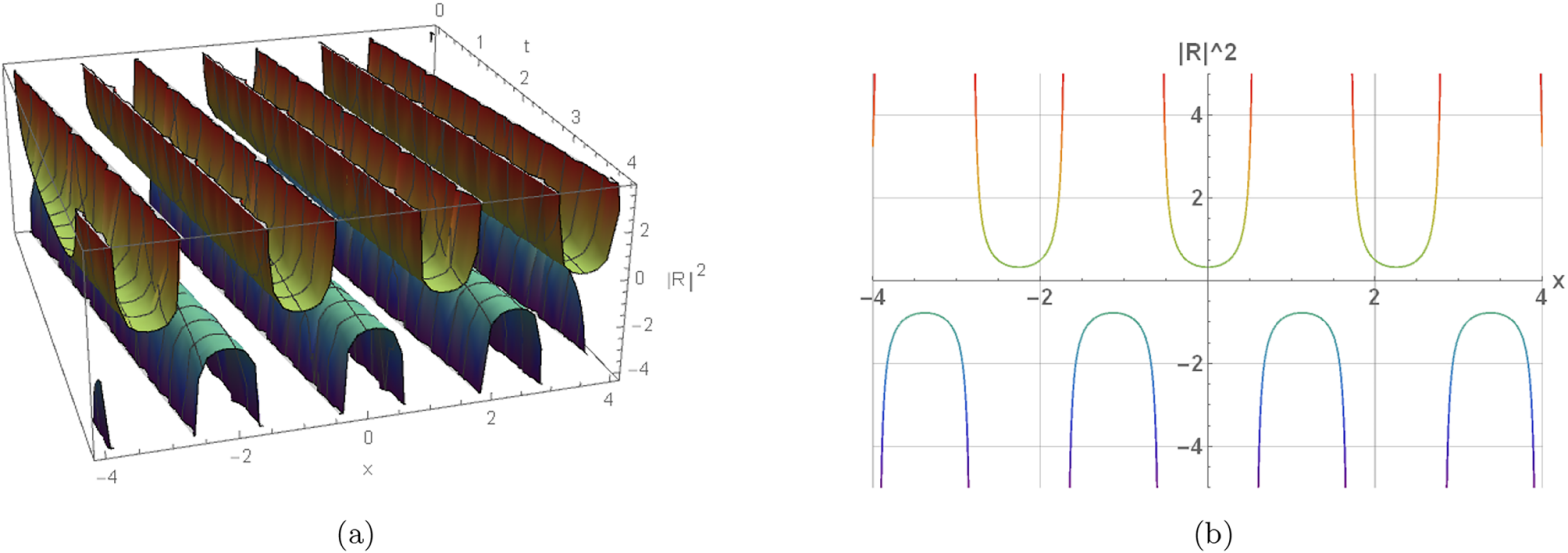
Graphical representation of a singular periodic solution of Eq. (17) by $$h_1=2, \omega =2, w_2=1.08, w_4=2, \eta _1=-2, \eta _2=-2, \eta _3=-2,$$$$\gamma =-0.625, y=0, z=0, n=2, s_2=1.315, s_4=1.495, \Pi _1=-1, \Pi _2=2$$.

**Fig. 4:**
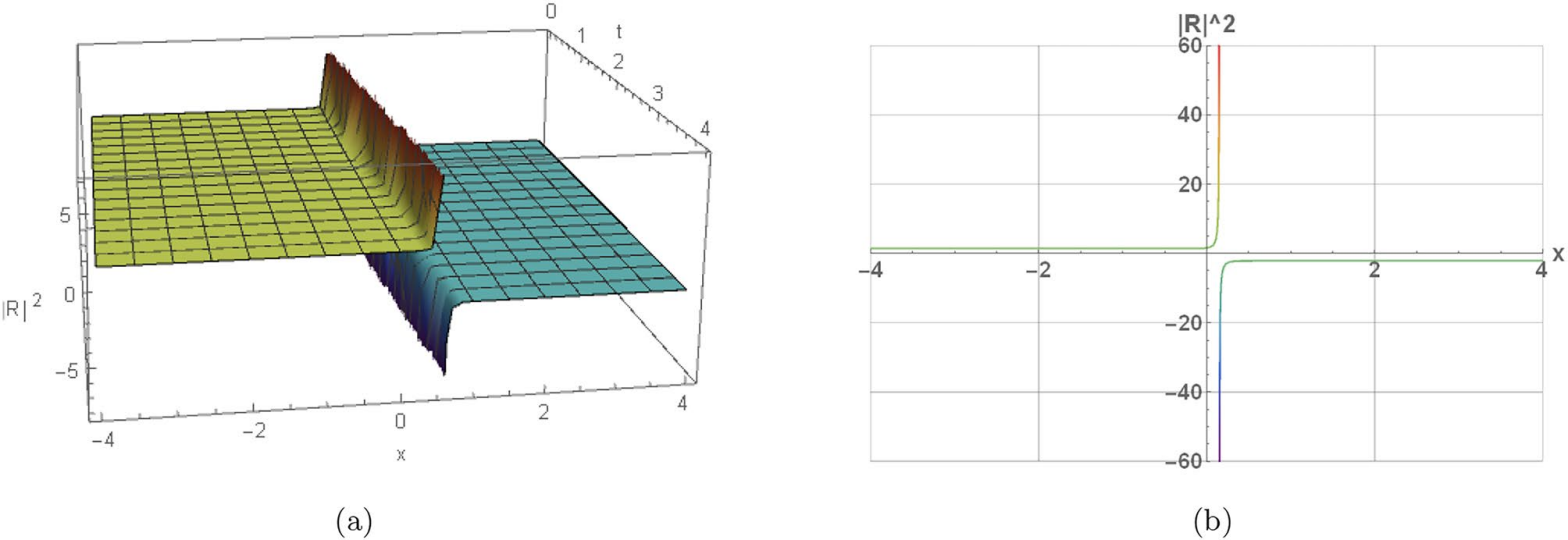
Graphical representation of a singular soliton of Eq. (18) by $$j_1=0.785, \omega =-2, w_2=-2, w_4=-2, \eta _1=-2, \eta _2=-2, \eta _3=-2,$$$$\gamma =-0.225, y=2.18, z=-2.33, n=2, s_2=-2, s_4=-2, \Pi _1=0.8, \Pi _2=-2$$.

## Discussion

In this section, we elaborate on the physical implications of the derived solutions. The bright soliton in Fig. 1 represents a localized pulse of energy that maintains its shape during propagation, a result of the exact balance between dispersive spreading and nonlinear self-focusing. The dark soliton in Fig. 2 corresponds to a stable localized structure that smoothly links two distinct background states, a hallmark of phase-shifted nonlinear wave interactions. The singular periodic solution shown in Fig. 3 describes a repetitive wave profile in space or time that contains points of divergence within each cycle, reflecting potential model breakdowns under extreme parameter regimes. Similarly, the singular soliton in Fig. 4 retains localization but exhibits unbounded amplitude or derivative values, indicative of strong self-focusing or excessive energy accumulation. These singular solutions emphasize inherent instabilities and mark the limits of the model’s physical validity. Collectively, the findings highlight how higher-order nonlinear and dispersive effects influence waveform formation, stability, and transitions. They further shed light on underlying mechanisms such as self-steepening, amplitude-dependent dispersion, and modulational instability, which are fundamental in nonlinear optics, plasma systems, and Bose–Einstein condensates.

## Linear stability analysis

To investigate the modulational (linear) stability of the system, we consider small perturbations around a plane wave solution^44^. The governing equation is

34 $$\begin{aligned} \begin{aligned} i R_t&- m_1 R_{xx} - m_2 R_{yy} - m_3 R_{zz} - 2 m_4 R_{xy} + 2 m_5 R_{xz} + 2 m_6 R_{xy} \\&+ \left( s_1 |R|^{-n} + s_2 |R|^{-2n} + s_3 |R|^{-3n} + s_4 |R|^{-4n} + w_1 |R|^{n} + w_2 |R|^{2n} + w_3 |R|^{3n} + w_4 |R|^{4n} \right) R = 0, \end{aligned} \end{aligned}$$

where $R(x,y,z,t)$ is a complex field, and $m_j, s_j, w_j$ are real parameters. The nonlinear potential is denoted as

$$V(|R|) = \sum _{j=1}^4 \left( s_j |R|^{-jn} + w_j |R|^{jn} \right) .$$

We analyze stability by considering the perturbed solution

35 $$\begin{aligned} R(x,y,z,t) = e^{-i p t} \left( q(x,y,z,t) + i u(x,y,z,t) + p \right) , \end{aligned}$$

where $p > 0$ is the constant background amplitude, and $q(x,y,z,t), u(x,y,z,t) \ll 1$ are real-valued perturbations.

### Linearization

Substituting (35) into (34), and retaining only linear terms in $q$ and $u$, we obtain the linearized system:

36 $$\begin{aligned} u_t&+ m_1 q_{xx} + m_2 q_{yy} + m_3 q_{zz} + 2(m_6 - m_4) q_{xy} - 2 m_5 q_{xz} + \left( V(p) + p V'(p) \right) u = 0, \end{aligned}$$

37 $$\begin{aligned} -q_t&+ m_1 u_{xx} + m_2 u_{yy} + m_3 u_{zz} + 2(m_6 - m_4) u_{xy} - 2 m_5 u_{xz} - \left( V(p) + p V'(p) \right) q = 0, \end{aligned}$$

where

$$V'(p) = \sum _{j=1}^4 \left( -j n s_j p^{-jn-1} + j n w_j p^{jn-1} \right) .$$

### Dispersion relation

To assess stability, we consider Fourier-mode perturbations of the form

$$q(x,y,z,t), u(x,y,z,t) \sim e^{i(k_x x + k_y y + k_z z - \omega t)}.$$

Applying this ansatz to equations (36)–(37), we derive the dispersion relation:

38 $$\begin{aligned} & \omega ^2 = \left( m_1 k_x^2 + m_2 k_y^2 + m_3 k_z^2 + 2(m_6 - m_4) k_x k_y - 2 m_5 k_x k_z \right) ^2 +\nonumber \\ & 2 p V'(p) \left( m_1 k_x^2 + m_2 k_y^2 + m_3 k_z^2 + 2(m_6 - m_4) k_x k_y - 2 m_5 k_x k_z \right) \end{aligned}$$

### Stability criterion

The system is linearly stable if $\omega ^2 > 0$ for all wavenumbers. Instability arises when $\omega ^2 < 0$, indicating exponential growth of perturbations. Thus, the sign of $V'(p)$ plays a critical role in determining stability:

- If $V'(p) < 0$, the system is generally stable.
- If $V'(p) > 0$, there exists a range of wavevectors for which modulational instability may occur.

### One-dimensional dispersion relation

To further illustrate the modulational stability, we consider a one-dimensional slice of the dispersion relation by fixing $k_y = k_z = 0$. In this case, the dispersion relation simplifies to

39 $$\begin{aligned} \omega ^2(k_x) = \left( m_1 k_x^2 \right) ^2 + 2 p V'(p) m_1 k_x^2. \end{aligned}$$

This reduction helps isolate the role of the longitudinal wave number $k_x$ and allows for direct visualization.

**Fig. 5:**
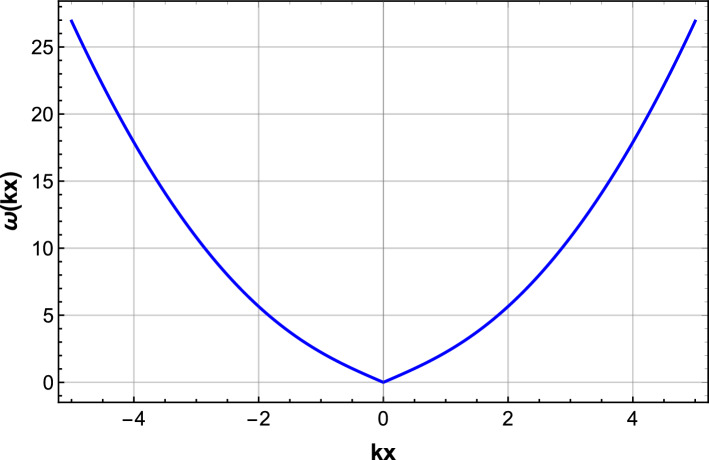
Dispersion relation $\omega (k_x)$ for fixed $k_y = k_z = 0$ in the stable regime. The curve is symmetric and strictly positive, confirming modulational stability of the plane wave background.

We plot the resulting dispersion relation $\omega (k_x)$ in Fig. 5, where we consider a focusing nonlinearity with parameters $s_j = 0$, $w_1 = 1$, $w_2 = 0.5$, and $w_3 = w_4 = 0$, ensuring that $V'(p) < 0$. The background amplitude is taken as $p = 1$, and the dispersion coefficient is $m_1 = 1$.

The linear stability spectrum provides direct information about how small perturbations evolve on top of the plane-wave background. If $\omega ^2(\textbf{k}) > 0$, the frequency $\omega$ is real and the perturbations are purely oscillatory, indicating stability. In contrast, if $\omega ^2(\textbf{k}) < 0$, the frequency becomes purely imaginary, which corresponds to exponential growth of perturbations and thus modulational instability. Therefore, the spectrum identifies both the stable oscillatory modes and the possible unstable modes together with their growth rates, and its graphical representation offers a direct means of assessing stability. Figure 5 illustrates the linear stability spectrum for the one-dimensional reduction. The plot shows how the perturbation frequency $\omega (k_x)$ depends on the longitudinal wavenumber $k_x$. Since the curve is symmetric, smooth, and strictly positive, all perturbation modes are oscillatory with no imaginary branches, which means that no modulationally unstable modes are present. This graphical representation therefore provides a direct confirmation of the stability of the plane-wave background in the considered parameter regime.

## Conclusion

**Figure 6:**
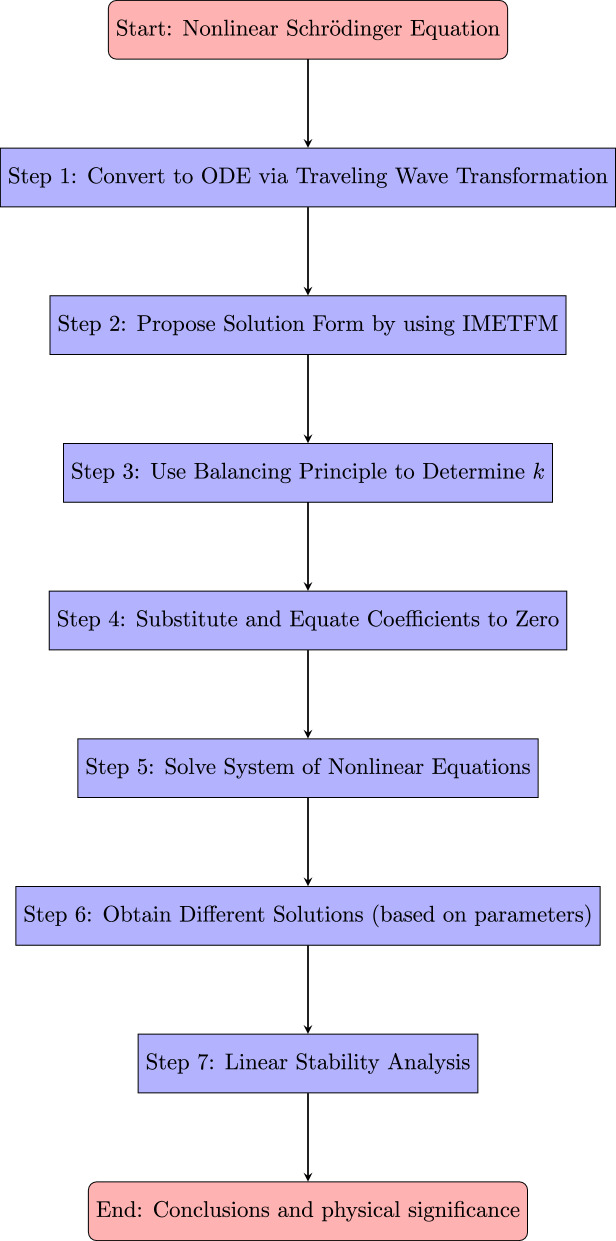
Flowchart illustrating the workflow of the present manuscript.

In this study, we investigated the (3+1)-dimensional NLSE with a generalized Kudryashov-type self-phase modulation and successfully derived novel soliton solutions using the IMETFM. The analysis revealed a wide spectrum of solitary wave structures, including bright, dark, and singular solitons, whose properties were strongly influenced by the interplay between higher-order nonlinearities, dispersive effects, and the parameters associated with the generalized self-phase modulation term. These analytical solutions provided deeper insights into the complex dynamics of nonlinear wave propagation in physical systems such as optical fibers, plasma environments, and Bose–Einstein condensates. Moreover, the effectiveness of the IMETFM in handling higher-dimensional nonlinear evolution equations was clearly demonstrated, establishing it as a reliable and versatile analytical tool. The conclusion of the workflow of the manuscript provided in the flowchart in Fig. 6.

For future research, this work could be extended by incorporating external perturbations, variable coefficients, or higher-order effects such as Raman scattering and self-steepening, in order to capture more realistic physical scenarios. In addition, direct numerical simulations are recommended to validate the stability and applicability of the analytical solutions under practical conditions. Finally, exploring fractional-order versions of the NLSE and examining their soliton dynamics may open promising avenues for advancing the understanding of complex nonlinear phenomena.

The methodological workflow for this manuscript is shown in the flowchart below. This figure shows the study’s logical flow from problem definition to the application of the suggested approach and the acquisition of the study’s conclusions.

## Data Availability

The datasets used and/or analyzed during the current study are available from the corresponding author upon reasonable request.
